# Integrating COM-B and the person-based approach to develop an ACT based therapy programme to raise self-determination in adolescents with obesity

**DOI:** 10.1186/s12913-023-09930-6

**Published:** 2023-10-26

**Authors:** Jennifer S. Cox, Aidan Searle, Gail Thornton, Julian P. Hamilton-Shield, Elanor C. Hinton

**Affiliations:** 1 National Institute for Health Research Bristol Biomedical Research Centre Diet and Physical Activity Theme, University Hospitals Bristol NHS Foundation Trust and University of Bristol, Bristol, BS8 1TU UK; 2 Ms Gail Thornton, Patient & Public Involvement Representative, Bristol, UK

**Keywords:** ACT, Obesity, Adolescents, Clinical, Self-determination, Behaviour change

## Abstract

**Background:**

This paper details the development of the Adolescent Intrinsic Motivation ‘AIM2Change’ intervention to support weight-management in young people previously unable to make changes whilst attending a tier 3 weight management service for children and young people. AIM2Change is an acceptance and commitment therapy based intervention that will be delivered one-to-one online over a seven-week period.

**Methods:**

To develop this intervention, we have triangulated results from a qualitative research study, patient and public involvement groups (PPI) and a COM-B (capability, opportunity, motivation, behaviour) analysis, in a method informed by the person-based approach.

**Results:**

The integrated development approach yielded a broad range of perspectives and facilitated the creation of a tailored intervention to meet the needs of the patient group whist remaining pragmatic and deliverable.

**Conclusions:**

The next steps for this intervention will be in-depth co-development of the therapy sessions with service users, before implementing a proof of concept trial.

**Supplementary Information:**

The online version contains supplementary material available at 10.1186/s12913-023-09930-6.

## Background

Obesity in childhood is associated with a wide range of adverse health outcomes, both during childhood and across the life-course, including an increased risk of diabetes [1], heart disease [2] and 16 different types of cancers [3]. For those who experience obesity in childhood, their weight status often tracks into adulthood [4, 5]. The NHS cost of treating obesity and its associated illnesses reached £6.1 billion in 2014/15 and is increasing year-on-year [6]. It is therefore in the interests of both the NHS and the individual to develop innovative ways that support every patient within a weight management service.

Overweight and obesity are frequently approached with the incomplete concept of ‘eat less and move more’ [7], which could be perceived to create a calorie deficit resulting in weight loss [8]. However, this vastly underestimates the complex, biological, environmental, social and psychological drivers to eating [9, 10]. Maintaining health behaviours and sustaining weight loss requires cognitive control, motivation and self-regulation [11], particularly when the environment does not support health [10, 12]. When considering the numerous biological drivers that oppose weight loss for evolutionary survival reasons [13], including the Hypothalamic Pituitary Axis, leptin and ghrelin that work to maintain food intake and energy stores, losing weight is difficult, and maintaining weight-loss is a potentially bigger challenge [14]. Only a small percentage of people who do lose weight sustain the loss, with many regaining all they have lost [14, 15].

Children with severe obesity may be referred to a tier-three, hospital-based, multi-disciplinary team clinic [16]. The Care of Childhood Obesity clinic at Bristol Children’s hospital was used as a model to develop the new Complications related to Excess Weight clinics, which will go some way to addressing inequalities of access [17]. These clinics treat patients experiencing complex and enduring obesity, with a body mass index (BMI) above the 99.6% percentile for age and sex, and health conditions resultant from excess weight., Patients seen at the clinic are treated using lifestyle guidance, dietetic programmes and psychological support, from a multi-disciplinary team. Following assessment of the severity of obesity in presenting patients, the clinic can utilise diagnostic and genomic testing, pharmaco-therapy and bariatric surgery.

However, the two reviews of the clinic to date acknowledged that the approaches used did not suit everyone. Younger patients without a family history of obesity [18] and with the advantage of motivated and more resourced families, are most likely to benefit from the clinic’s approach [19]. For the year 2021/22, 46% of patients were not supported by the clinic to improve their adiposity and this patient group was found to have complex obesity, with related behavioural or social issues including autism spectrum disorder, attention deficit hyperactivity disorder or socioeconomic deprivation. These patients may drop out of the clinic or may continue to attend but without achieving the desired outcomes [18].

To support long-term success, the implementation of evidence-based behaviour change strategies are recommended [20]. An understanding of the complex and numerous factors that cause and maintain obesity can be used to implement targeted behaviour change [21]. To be effective, the development of the intervention should be theory-led and grounded in behavioural science, but also incorporate the patient perspective [16, 20]. Such integrated approaches to intervention design have been shown to offer enhanced clarity of intervention focus and ability to meet patient needs [22].

The objective of this research is to develop a feasible, effective intervention tailored for those young people who do not experience progress in a tier-three weight management service. Here, we present the first three stages of this process: (1) intervention planning, (2) intervention development and (3) testing the concept. This intervention was initially planned and designed at the Bristol Care of Childhood Obesity clinic, with the view to trialling the intervention in the Complication from Excess Weight NHS clinics [17]. Thus, the scalability and deliverability of the intervention across sites providing broad geographic and demographic reach has been considered in this context.

## Approach

To select frameworks of behaviour change, a recent systematic review of behavioural intervention development tools was consulted [20]. Methods from (i) the person-based approach [23] facilitating a focus on patient acceptability and feasibility, and (ii) a theory and evidence-based approach (the COM-B [24]), were brought together for intervention development. The stages were conducted in an iterative manner, with each step informing the next. The intervention planning stage included re-analysis of existing data through qualitative methods. The intervention development stage included the design of the intervention using the COM-B framework and the development of guiding principles [23]. Importantly, patient and public involvement (PPI) groups were then consulted to ensure the intervention met the needs of the population.

To ensure lived experience remains the primary focus of our intervention design, a member of the PPI group, with lived experience of obesity (GT), was invited to join the research team as a PPI representative. Her contributions iteratively feed into each stage of the development.

### Stage 1: Intervention planning

#### Qualitative methods

##### Rationale

Understanding the population for whom the intervention is being designed is a fundamental first step [25]. A service review was first conducted over ten-years ago [19], and so the review was repeated in 2019 [26]. A high reliance on the support of the clinical team was found, with desire for more intensive medical involvement in the weight-loss process, with low levels of patient belief that they had the capability to sustain change themselves. Parallels with the three components of self-determination theory were drawn, as the patients did not demonstrate fulfilment of relatedness (feeling supported), autonomy (empowered to make their own choices), or a sense of competency (feel able to make changes) [27–29]. Without self-determination, which includes being attuned with our intrinsic motivations (in this case, internal reasons for weight change), weight-loss relies on external factors. Indeed, patients appeared not to utilise intrinsic motivations, meaning there is a potential for improving weight-loss outcomes if these components are increased. Evidence suggests that lifestyle changes that are intrinsically rewarding to the individual in and of themselves, are more likely to be sustained [30, 31]. The findings from the service review suggested that if self-determination can be increased in this population, it not only offers opportunity for improved weight-management outcomes but also facilitates improved self-management skills that can be transferable throughout the young person's life. The rich qualitative data in the study [26] provided the opportunity for re-analysis from an intervention planning perspective that is presented here.

##### Qualitative research process

Semi-structured interviews were conducted with twelve families who attended the Care of Childhood Obesity clinic [26]. The interviews were open to all service users, however all but one of those who took part were not currently seeing changes to their weight or co-morbidities following the current service approach. The views of the service user achieving weight-loss were removed from this analysis as data saturation in this area was not met. Interviews were conducted via telephone and audio recorded between January and August 2019, except one which was conducted in person. The interview data were thematically analysed following the six-step procedure of Braun & Clarke [32] by two independent and experienced qualitative researchers (JC and AS). For this intervention planning process, the transcripts were re-visited to extrapolate useful insights on the pragmatic and logistical aspects of the clinic experience, which were not focused on in the previous publication [26].

##### Findings

Our additional qualitative extrapolation of the interviews focussed on two themes that were framed with regard to (1) access to the clinics and the perceived support provided by clinic staff and (2) thoughts on group-based interventions provided by the clinic.

Pragmatically, the service user group were clear in reporting the difficulties of in-person attendance at this clinic, with travel, missing school and the cost being significant barriers (Table 1; Part 1). The interviews were conducted prior to the Covid-19 pandemic and the concept of online services was considered tentatively. The service users’ perspective on group work was more nuanced, with some in favour of the peer-support offered by group work and others preferring the confidentiality of one-to-one sessions due to the sensitivity that young people and peers may experience during adolescence (Table 1; Part 2).

**Table 1:** Additional quotes from qualitative interviews with service users and parents

**Part 1: When asked about access to the current clinic, and the prospect of additional support that could be offered at the clinic …**	
Young person, aged 16	Nah, just every single appointment ever, they [school] just start having a go. I haven’t had that many days off, like if I am ill. Most of mine are like appointments. I can’t really control how many appointments I have.
Parent of girl, aged 16	She just doesn’t like having to take the whole day off if we’ve got to get the bus from school and that cos it affects her attendance but um, they’re aware of what it is cos it’s a letter, and they know I don’t drive at the minute as well.
Parent of boy, aged 15	I am not trying to be difficult, I am in the process of – I have just applied for PIP. [personal independence payment] I should have done it years ago. Things may start to get a little bit easier in terms of parking, transport, so it could become easier. But yes, it could be [unfeasible to come to additional sessions]. But that’s because it’s expensive, not because I am not happy to travel to the city, I am very happy to travel to the city, it’s just practically … it’s very difficult at the moment.
Parent of boy, aged 14	No, it’s not far, we are only by [district] but in the morning, it is far. It’s stationary traffic, crawling in traffic all the way to the hospital. So it can take any time in the car. I came in yesterday by taxi cos my husband was at work, and I was worried we wouldn’t find anywhere to park … and the taxi took 30 min, if I had come by myself, and then tried to drive around trying to find somewhere to park, potentially not being able to park, park in the centre and walk up, um, it takes even longer.
Parent of boy, aged 14	It depends on when they are … I don’t really want to take him out of school any more if I don’t have to. He has done this, with Alive and Kicking [tier two weight management service in the area] we did do their six-week programme, but I don’t know if we would do that now. You know. It just depends on the timings really. If it is at the children’s hospital, it is all the hassle of getting in there again.
Parent of boy, aged 15	The distance is too long really, he ends up, the school won’t be happy. He ends up with a whole day off school each time.
Parent of boy, aged 15	It’s difficult because I have quite significant mobility issues myself so coming to [the city] is really difficult, really tiring and risky because for me the more I walk, the more likely it is I end up in hospital with a chronic bacterial infection due to complex swelling. I have a complex chronic health condition myself. To be honest, by the time I am going home I am exhausted, in pain and just wanting to go home. But it takes a few hours, to wait for the hospital bus that has been cut back and cut back and you can wait for an hour for the bus to come, and then, it’s just, it’s difficult. It costs a lot of money, even though I can claim the cost of the train back I have to pay for parking at the station, I have to pay for the fuel to get to the station, there is no public transport to the station and I can’t walk. You know, it’s quite a mission to come, so by the time we are going home, I am just desperate to go home.
Parent of boy, aged 13	I wonder if there is a way, I guess it may be, it might be a difficult area to go into, but whether there was a way of doing something positive via an online platform, could get support, encouragement and be linked up … but it would have to be safe but, but um, whether there is a hope of doing something like that? I think [child] would feel more safe (sic) and be more open about how he is feeling and that, than face-to-face. At this stage in teens, in the middle of puberty, at an awkward stage in terms of making new friends and feeling confident about yourself … [interviews conducted prior to Covid-19 and the increase in online platform use for appointments and schooling]
**Part 2: When asked their thoughts on group-based interventions**	
Parent of girl, aged 13	Um …. No, I am not sure, cos she doesn’t like to … she is familiar with the dietician and social worker, but she doesn’t like to talk about things …
Parent of boy, aged 14	At this stage in teens, in the middle of puberty, at an awkward stage in terms of making new friends and feeling confident about yourself … But in terms of … he doesn’t have much confidence left after the mental health problems he has had over the last … two years now, he doesn’t have much confidence left for the new friends at the moment.
Parent of boy, aged 14	I think the group thing would be quite good, and might be better, seeing other families and how they do. Oh, that is the other thing, [child] wouldn’t be, it would be important to be with the ages. In the one we went to before, he was 11 and we were pushed into the older group, rather than being with the littler ones, would this be run by age group?
Young person, aged 16	Well, that is worse isn’t it [than speaking one-to-one with clinicians] … I get that other people would be in the same boat as you but … I think when it comes to group sessions … I think stuff like that is a bit personal, and not everyone is in the same boat. Like they may think “Yeah, they’ve got exactly the same thing”. No. Cos each person needs the people to kick for them.
	…. The younger ones no, but like 15plus then that could be like … more helpful. Helpful to comprehend what they are saying and what is helpful to take away from it. Cos with the doctors it is just facts, facts, facts, facts. They try to help but they are not in the situation. Whereas if it was an older group, you could be like “Yeah, I have tried this before, it doesn’t exactly work but if you change a few things …” like that. We could all help each other.
Parent of girl, aged 13	One-to-one Is better. She doesn’t like – like I said, she is quite personal. She’ll get upset otherwise and I don’t want her to feel like she doesn’t want to – not want to come back cos of that reason. I’ve just got her on a level where she is comfortable talking to certain people. Whereas if it was a group of people that she doesn’t- she might lose her temper a little bit, and I don’t want her to do that or get upset, or go home crying, or have a negative – that might have a negative feel on her. Me, it wouldn’t be too bad cos other parents might have other ideas but, for her it probably would help her …. Yeah, if we change it—I don’t want to rock it, I’ve just got her in – it’s taken me a year to get her where she is so …

### Stage 2: Intervention development

#### COM-B

##### Rationale

Whilst knowledge about the behaviours we should be increasing (e.g. activity), and those we should be decreasing (e.g. fast food consumption) is important, on its own this knowledge is rarely sufficient to create sustained change in behaviour [33]. Research into the psychology of behaviour change suggests that to be able to sustain changes, the individual needs to have the Capability, Opportunity and Motivation to perform the new Behaviour [24]. This COM-B offers a framework on which to design interventions with these key facets of behaviour change in mind [23, 34].

##### Approach

Using the rigorous COM-B framework for intervention design [23], an in-depth behavioural analysis was conducted to understand what needs to change before the target group (in this instance a clinical paediatric population with obesity) can change the target behaviour (in this instance, self-determination). The analysis is structured through the completion of a series of COM-B worksheets, each focussing on a different aspect to help determine how the behaviour change could be brought about. The final stages of the COM-B model guide the content and intervention implementation options and involve understanding which of 93 behaviour change techniques (BCTs), would be effective in bringing about change and via what delivery mode. Throughout, the model utilises the APEASE (Practicability, Effectiveness, Affordability, Side-effects, Equity) framework to ensure feasibility of the selected intervention. Three authors (JC, EH & AS) independently completed the COM-B process worksheets then met with clinical expert (JHS) to finalise decisions and create an overarching document (Supplementary materials).

Consideration was then given to evidence regarding the extent to which existing psychological therapies can achieve the behavioural change techniques identified from the COM-B process. Three authors (EH, AS and JC) reviewed the evidence to determine what interventions were currently being used to elicit self-determination and intrinsic motivation, in the context of weight management.

##### Findings

*COM-B stage 1*

It was apparent that an intervention to raise service users’ sense of self-determination could be successfully achieved by targeting psychological capability, social opportunity and reflective motivation or automatic motivation (Supplementary materials, Worksheet 4).

*COM-B stage 2*

The intervention could be effectively delivered via education, modelling, training, or enablement pathways (Supplementary materials, Worksheet 5). If the intervention were to look at changing policy, the policy categories that could be applicable were to look to influence guidelines and service provision (Supplementary materials, Worksheet 6). Based on these results, and the perspective of a clinical expert (JHS) and PPI, it was agreed that a training approach was more in-keeping with ACT’s standpoint of working alongside service users to develop collaborative solutions, rather than taking a teaching approach. It was also agreed that the clinical team modelling desired behaviours, to demonstrate how a service user could embody the behaviour change, would be an appropriate technique.

*COM-B stage 3*

To establish which behaviour change techniques (BCTs) were helpful to include, the BCTs were considered through the lens of increasing self-determination and were chosen based on their ability to support self-determination and intrinsic motivation. Worksheet 7a (Supplementary materials) documents all the beneficial BCTs, which include behavioural practice/rehearsal (for example practising mindfulness when feeling calm, for the skill to be more readily available in times of stress), and valued self-identity (affirming the person’s self-identity in line with the behaviour change). Furthermore, a tailored list of specific motivational BCTs (MBCTs) has been developed by expert consensus, including the authors of the COM-B model and self-determination theory. It therefore seemed pertinent to also include these MBCTs in the intervention, with items such as using empathic listening, clarifying expectations, and dealing with pressure being highly relevant (Supplementary materials, Worksheet 7b).

For clarifying modes of delivery, COM-B identified face-to-face or phone to be potential delivery options, particularly by video-call (Supplementary materials, Worksheet 8). The qualitative interviews demonstrated the difficulty patients have in accessing the clinic due to its city-centre location and the costs this incurs (Table 1), therefore we opted to run the programme via video-call.

#### Review of interventions to increase intrinsic motivation

While Cognitive Behavioural Therapy (CBT) is often the default in weight-management [35], there is a developing evidence base in favour of Acceptance and Commitment Therapy (ACT) [36]. ACT is a third-wave cognitive behavioural intervention, that utilises core processes that support emotional regulation, including acceptance, mindfulness, and values-based work to deepen intrinsic motivation [37]. ACT is therefore recognised as an appropriate psychological therapy for increasing self-determination [38, 39]. ACT may offer superiority to CBT in the domain of raising self-determination, due to its ability to enhance emotional self-regulation through its focus on self-awareness and mindfulness. Whilst other third-wave therapies including Mindfulness Based Cognitive Therapy and Dialectical Behavioural Therapy may also offer opportunities to raise self-determination, ACT currently demonstrates the greatest efficacy within weight management [40].

Recent reviews have considered ACT as an important approach to treating obesity in adults [11, 41–44]. Indeed, ACT has been adopted in a range of adult weight management services [42, 43], including the recent Supporting Weight Management (SWiM) trial [45]. Importantly, several iterations of ACT have been successfully developed specifically to work with young people, children, and adolescents [46, 47]. Following the above review, the present authors are now conducting a formal scoping review on the use of ACT for weight management in young people, details of which are currently available in the pre-registration (https://osf.io/523du/), and will be presented elsewhere when complete.

#### Guiding principles

##### Rationale

The development of guiding principles helps to clarify an intervention’s objectives and ensure the design meets the needs of the end user. Throughout intervention iterations, the guiding principles should be consulted to ensure the intervention retains its focus.

##### Approach

**Fig. 1:**
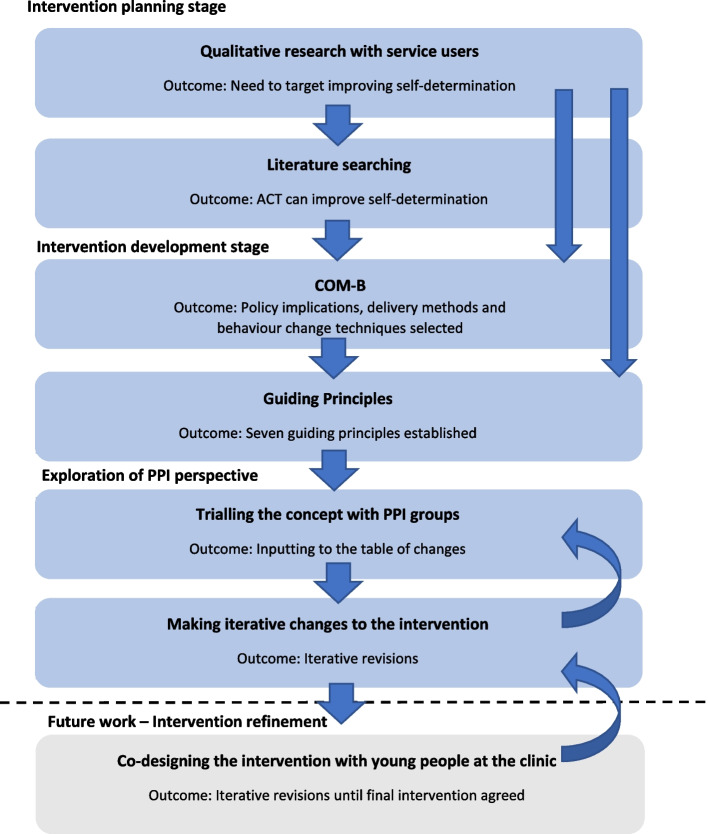
Stages of intervention development

To develop the guiding principles, a person-based approach was utilised [24]. Together, the knowledge gained from the COM-B behavioural analysis, qualitative research study, review of the evidence and the PPI groups was considered, and overarching objectives of the intervention were agreed on by the research team. Key features of the intervention that will ensure each principle is achieved are detailed in Table 2. The development of guiding principles and key features is iterative (Fig. 1), as the intervention continues to evolve in response to PPI feedback and feasibility work with the clinical population, so it is likely that these principles will evolve too.

**Table 2:** Guiding principles

Guiding principles	Key features of the intervention that will ensure the principle is achieved
The interventions must be designed specifically for this population	• PPI work and voices of those with lived experience will be central to the design
	• Interventions designed for adults are not directly applicable to children [48, 49], therefore this intervention will be specifically designed
The Interventions should not create dependency on care, and instead help develop patients’ autonomy	• The intervention will work to develop patients’ sense of self-determination through the facets of enhanced competency, autonomy, and relatedness
	• The clinical team and the families will adopt a supportive role, encouraging the young person to take responsibility for change
	• The balance of responsibility will be communicated clearly from the outset
Development of open & trusting relationships is important	• Time will be taken to build rapport and create a trusting, warm environment
	• Open communication will be encouraged throughout the process
Interventions should not increase pressure on parent/child relationships, and should instead support this sometimes-difficult relationship	• The service review highlighted how tensions within some families were exacerbated by disagreements around weight Cox et al. [26]. This intervention seeks to support parents to enable their children to lead the changes, which has been shown to decrease conflict
	• Young people can choose when they would like their parent/guardian present during a session, whether they would like a different support person to attend, or whether they would like to attend alone. This choice may change from session-to-session
The intervention must consider the whole person and not just issues regarding weight	• The intervention will treat eating behaviours in the context of the young person’s life and experiences
	• All changes will be selected for their ability to fit within the context of the young person’s life, in order for them to be sustainable
	• The intervention will offer transferable skills that the young person can utilise in other aspects of their life
The intervention must be accessible	• The programme will be delivered online to facilitate access. This avoids travel time and parking costs
	• Sessions can be scheduled at times to avoid missing school/parents missing work
	• Funding will be allocated for data allowance to ensure access to video-calling, and tablets can be lent to anyone without access to a smartphone or computer
	• For those without a private place to speak at home, alternative arrangements will be supported
Interventions should aim to target long-term, sustainable lifestyle change, not offer a quick fix	• Focus will be on changes that can be maintained
	• Intervention will be tailored to work with the context of each participant’s life
	• The intervention will include meta-cognitive awareness of long vs short term outcomes of our decisions
	• Young people will make the decisions as to the changes they are making, meaning they will be more appropriate than a one-size-fits-all approach

##### Findings

The guiding principles (at point of publication), and the key features of the intervention that will help the principles be achieved, are detailed in Table 2.

### Stage 3: Testing the concept and iterative development

#### Patient and Public Involvement

##### Rationale

To ensure the programme works for the end-user, PPI is vital throughout the intervention process.

##### Approach

Based on the planning and development work described above, a protocol ACT therapy manual was developed by a health psychologist trained in ACT (JC). The concepts were discussed with four PPI groups: one with young people of healthy weight and two with adults with obesity who had experienced obesity during their childhood and adolescence (some of these participants’ children were also currently experiencing obesity). The fourth group were young people with obesity who were currently engaged with a tier-3 weight management programme (Table 3).

**Table 3:** PPI participant details

Group	N	Demographic
Group 1	5	Young people aged 14–17 years
Group 2	5	Adults aged 18 + with lived experience of obesity, including obesity in childhood/adolescence, some with their own children who are currently experiencing overweight and obesity
Group 3	4	Adults aged 18 + with lived experience of obesity, including obesity in childhood/adolescence, some with their own children who are currently experiencing overweight and obesity
Group 4	6	Young people aged 12 – 17 years, currently experiencing obesity

##### Findings

The feedback of the PPI group members has actively influenced both the content and the delivery of the intervention. This rich and significant feedback, and how it has been incorporated into the development process, is documented in a Table of Changes (Supplementary materials).

The PPI groups were in favour of the focus on intrinsic motivation and taking an approach of working collaboratively, with many reflecting on their own experiences of failed diet attempts when driven by external reasons, and the negative impact this pressure has had on them. Participants perceived this potential intervention as giving them a new perspective on weight management (“a new way to consider this”), whilst resonating with their own experiences (“I feel like you described my teenage years”). Changes were made to the structure of sessions (see Table of Changes, Supplementary materials), with young people now being given the option as to whether their parent, or another support figure, attends sessions with them or not. The use of the term ‘mindfulness’ was also challenged due to the term “constantly being thrown at us at school”. Overall, the group considered the approach holistic, where the therapist would seek to “build a relationship with them beyond their weight”. The transferability of the skills involved offered them “help for life”, rather than a programme that was purely about weight loss.

## Discussion

Through integrating person-based insight, theory and evidence, an ACT based approach to raise self-determination in paediatric weight management has been devised. The approach will be further co-developed with young people in the Care of Childhood Obesity clinic, before a proof of concept trial is conducted within the Care of Childhood Obesity clinic and two independent Complications of Excess Weight clinics [50], with the view to incorporating this intervention into all such clinics in the future.

The integration of techniques from COM-B [23] and the person-based approach to intervention design [24], and the contribution of a PPI representative (GT) within the research team ensures lived-experience is fully integrated within this intervention design. This enables the intervention to meet current NICE guidelines, which request that all paediatric weight management interventions “have taken into account the views of children, young people and their families” [16]. Contributions from the patients and their families led the decision to take the novel approach to target self-determination and intrinsic motivation, which we perceive to be the cornerstone to life-long change. The service user perspective has also heavily influenced the logistical aspects of the intervention. The four trials that have assessed ACT use in similar young populations have also shown promising feasibility results; however, none of the interventions have been developed in a service user-led way and all have included ACT as part of an integrated weight-management intervention with multiple elements, making it difficult to delineate the effectiveness and feasibility of the ACT components in this setting [51–54]. Practical factors such as access, location and timing contribute to the high attrition typically seen within weight-management interventions [55, 56]. Consequently, the proposed intervention follows the service user lead on how and when they would like to receive the intervention, and it is hoped will yield enhanced completion rates.

The evidence suggests that ACT processes, including supporting meta-cognitive thought, clarification of values and acceptance of difficult thoughts and feelings, enhance emotional and self-regulation [11, 39]. Our PPI feedback celebrated the holistic approach to care, which offers patients a new skill set that is transferable to other elements of their lives.

Whilst CBT is the default model for clinical care, theory suggests that ACT as a third-wave CBT therapy may offer enhanced ability to generate autonomous motivation, self-regulation and sustained change [11, 43]. Potentially this is via mechanisms including emotional regulation, non-judgemental awareness, and metacognitive thought [39]. As we are creating a novel, tailored intervention in this paediatric setting, this work is a crucial step to translating theory into clinical practice.

A further intervention development process will include delivering and interactively developing the seven-week programme, session-by-session, with young people from the Care of Childhood Obesity clinic. The programme will evolve iteratively based on participants’ qualitative feedback through ‘Think aloud’ interviews to result in a programme that is tailored to meet the needs of this population. When necessary, the guiding principles will also evolve based on participant feedback, together with the evidence base including the on-going scoping review [57]. To widen the diversity of the patient voice, ensuring ethnicity is considered, further diverse and inclusive PPI advisory groups will be held with the help of mutual support group, Obesity UK. Loan devices are being made available to ensure young people from underserved communities, without the required technology, can also contribute. Once developed, the intervention will enter a proof of concept trial study, prior to a full trial to test for effectiveness within the Complications of Excess Weight clinics.

Limitations within the methodology of this development work include having a limited number of PPI advisors who were within the target age and weight-status of the intervention. We were mindful not to overload the young people who attend the Care of Childhood Obesity clinic, as they had already been involved with the service review [26], another research trial not connected to this intervention [48], and crucially will be involved with the in-depth session-by-session development phase of this intervention. Therefore, we sought alternative groups of young people with relevant lived experience to contribute to this early PPI work.

In conclusion, this development paper details the integrative approach taken to establishing ‘AIM2Change’. With further evidence, theory and patient-led research methods iteratively contributing to the final intervention, we hope to have produced an intervention that is acceptable, effective, and adhered to when rigorously trialled.

### Supplementary Information

**Additional file 1.** 

## Data Availability

This work is supportive of open-research and all materials can be made available on request to Jennifer.cox@bristol.ac.uk.

## Abbreviations

ACT: Acceptance and commitment therapy
AIM2Change: Adolescent intrinsic motivation to change
CBT: Cognitive behavioural therapy
COM-B: Capability, opportunity, motivation, behaviour
NHS: National health service
NICE: National Institute for clinical excellence
NIHR: National Institute for health research
PPI: Patient and public involvement
