# Supplementary material for: Integrating COM-B and the person-based approach to develop an ACT based therapy programme to raise self-determination in adolescents with obesity
Source: BMC Health Serv Res. 2023 Oct 26;23:1158. doi: 10.1186/s12913-023-09930-6 (PMC10601199; doi:10.1186/s12913-023-09930-6)
Supplement: Supplementary file 1 — Additional file 1. [file 12913_2023_9930_MOESM1_ESM.pdf]

Using the COM-B to understand intervention need in the development of a paediatric clinical weight management intervention targeted at those young people who are not experiencing weight change from attending the clinic.

Fig 1. The behaviour change wheel with sources, intervention functions and policy categories

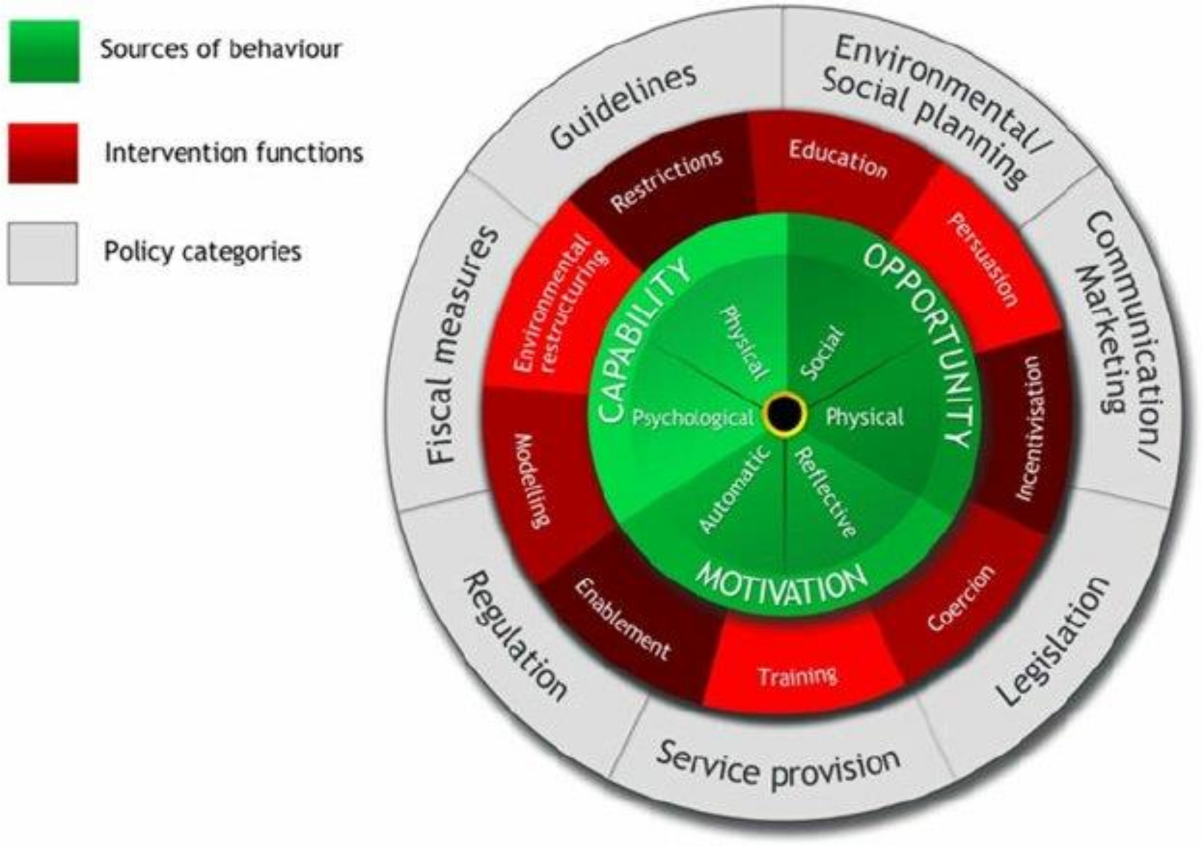

Fig 2. The process of completing the behaviour change wheel guide to designing an intervention.

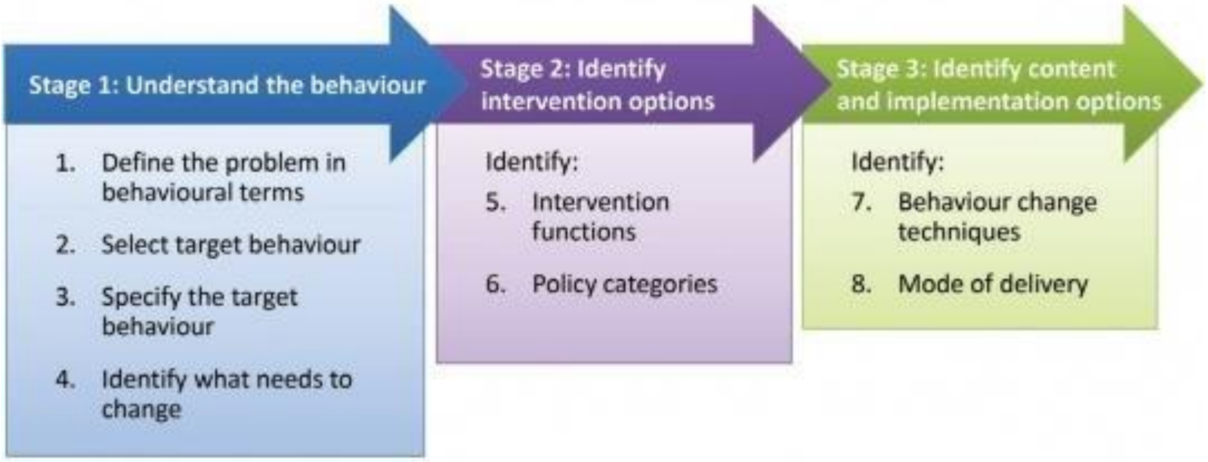

NB As the intervention aim has been established via qualitative interviews and literature searching, the COM-B process for this intervention begins at Stage 1. Step 4.

Stage 1. Step 4. Understanding the behaviour: Identify what needs to change.

Worksheet 4

**Using the COM-B model to identify what needs to change to enable young people to increase their intrinsic motivation.**		
**COM-B Component**	**What needs to happen for the target behaviour to occur?**	**Is there a need for change?**
Physical capability: our physical strength, skill or stamina	N/A	No
Psychological capability: our knowledge/ psychological strength, skills or stamina	Perseverance and stamina will be required for these patients as it's possible they will need to manage and monitor changes for a long period.<br><br>Psychological strength to make decisions based on long-term not short-term reward.<br><br>Increase in self-efficacy.<br><br>Psychological skills in identifying what their values and goals are.<br><br>Skills to appraise their environment and to understand how to operationalise this to their benefit (environmental restricting, cueing etc).<br><br>Skills to negotiate conflict.<br><br>Skills to negotiate sabotage by friends/family/etc.	Yes<br><br>Yes<br><br>Yes<br><br>Yes<br><br>Yes<br><br>Yes<br><br>Yes
Physical Opportunity: opportunities provided by the environment, such as time, location and resource	N/A	No
Social Opportunity: opportunities as a result of social factors, such as cultural norms and social cues	Young people need to be supported by an 'autonomy-supportive' environment and support style from parents and clinicians. Patients will need this form of support from their close family and friends to allow them the autonomy to take responsibility for their own behaviour. This will be a change in the power-balance	Yes

	for many families, where parents usually have the greater control over their child's eating.<br><br>Engaging peer support.<br><br>Parental modelling of intrinsic motivation and behaviour change driven from intrinsic means.<br><br>Challenging stigma and external drivers for change.	 <br><br><br>Yes<br><br>Yes<br><br><br><br><br>Yes
Reflective Motivation: reflective processes, such as making plans and evaluating things that have already happened	Patients will need to be prepared to evaluate their values and goals for this approach to work. They will need to develop skills in reflecting on their behaviour (both past and current) as the course goes on and making plans to take forward ideas from the clinic into their everyday behaviour.<br><br>Improved self-knowledge of what motivates oneself.<br><br>Reflection on past experiences and what helps/hinders in own life.	Yes<br><br><br><br><br><br><br><br><br><br><br>Yes<br><br><br>Yes
Automatic Motivation: automatic processes, such as our desires, impulses and inhibitions	Learning skills to inhibit automatic behaviours that allow short-term rewards and to favour a longer-term view, for example targeting the automatic processes that result in emotional/comfort eating.<br><br>Developing new behaviours as habits. This will support self-regulation as actions are less focused around conscious decisions. Healthy behaviours become embedded.	Yes<br><br><br><br><br><br><br><br><br><br><br>Yes

**Behavioural diagnosis of the relevant COM-B components**	Psychological capability; Social Opportunity; Reflective Motivation; Automatic Motivation

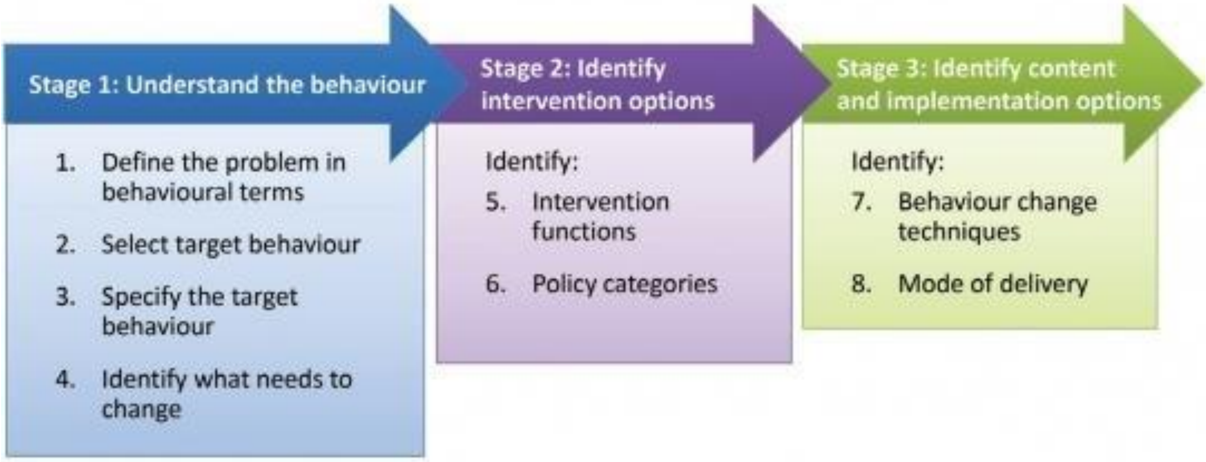

Stage 2: Identifying intervention content and implementation options.

Step 5. Identify intervention functions.

This stage involves using the results of the behavioural diagnosis carried out in Stage 1 to guide decisions regarding the content and delivery of the intervention. This involves first selecting suitable intervention functions (e.g. education, incentivisation) and policy categories (e.g. guidelines, legislation). (Fig. 1) Based upon these decisions, suitable BCTs are identified for inclusion in the intervention before finally deciding upon a suitable mode of delivery.

First, the intervention functions most suited to target the domains identified in the COM-B behavioural analysis carried out in stage 1 were selected using established links between COM-B and intervention functions (Table 1). The identified intervention functions suitable in the context of the target behaviours are then considered using APEASE criteria (affordability, practicability, effectiveness/cost-effectiveness, acceptability, side-effects/safety, equity) which allowed for evaluation of the appropriateness of the identified functions for incorporation into the intervention.

Table 1. Links between COM-B & intervention functions

INTERVENTION FUNCTIONS -->	EDUCATION	TRAINING	PERSUASION	INCENTIVISATION	COERCION	RESTRICTION	EBABLEMENT	ENVIRONMENTAL RESTRUCTURING	MODELLING
COM-B COMPONENTS									
Physical Capability		■					■		
Psychological Capability	■	■					■		
Reflective Motivation	■		■	■	■				
Automatic Motivation		■	■	■	■		■	■	■
Social Opportunity						■	■	■	■
Physical Opportunity		■				■	■	■	

Table 2. examples of intervention functions

Intervention function	Definition	Example of intervention function
Education	Increasing knowledge or understanding	Providing information to promote healthy eating
Persuasion	Using communication to induce positive or negative feelings or stimulate action	Using imagery to motivate increases in physical activity
Incentivisation	Creating an expectation of reward	Using prize draws to induce attempts to stop smoking
Coercion	Creating an expectation of punishment or cost	Raising the financial cost to reduce excessive alcohol consumption
Training	Imparting skills	Advanced driver training to increase safe driving
Restriction	Using rules to reduce the opportunity to engage in the target behaviour (or to increase the target behaviour by reducing the opportunity to engage in competing behaviours)	Prohibiting sales of solvents to people under 18 to reduce use for intoxication
Environmental restructuring	Changing the physical or social context	Providing on-screen prompts for general practitioner to ask about smoking behaviour
Modelling	Providing an example for people to aspire to or imitate	Using television drama scenes involving safe-sex practices to increase condom use
Enablement	Increasing means/reducing barriers to increase capability (beyond education and training) or opportunity (beyond environmental restructuring)	Behavioural support for smoking cessation, medication for cognitive deficits, surgery to reduce obesity, prostheses to promote physical activity

Worksheet 5

**Candidate intervention function**	**How would the intervention work in the context of improving a paediatric patient's intrinsic motivation?**	**Does the intervention function meet the APEASE criteria (affordability, practicability, effectiveness/cost-effectiveness, acceptability, side-effects/safety, equity) in the context of improving a paediatric patient's intrinsic motivation?<br>Consider this on the basis of this being an individual level intervention (not a population level intervention)<br><br>Explain whether or not each function is appropriate, including your rationale.**
Education	This function is appropriate as linked to 'Psychological Capability' – patients will need educating in psychological techniques and behaviours to increase their strength and stamina.<br>Psycho-education could be used to help support understanding of motivation and psychological pathways to success, self-understanding and evaluation, and planning for the future.	Affordability could be questioned given we're planning a 1:1 therapy, one hour a week for 7 weeks.<br>However, the intention is that this would reduce overall duration of time the patient is in the clinic. Targeted at current non-responders to the current treatment, so would likely go on to having complex health conditions/ NHS expense.<br><br>Using online platform (e.g. zoom) reduces cost. No clinic space required.

		The feasibility trial is designed to determine if it is acceptable.
Persuasion	No – this approach is too 'top-down' to be fitting with generating intrinsic motivation	N/A
Incentivisation	No - this approach is in contrast with generating intrinsic motivation (instead generating extrinsic motivation)	N/A
Coercion	No – this approach is too 'top-down' to be fitting with generating intrinsic motivation	N/A
Training	Yes – would be a fitting approach to impart skills for self-motivation and management, and for parents in creating an autonomy-supportive environment.	Affordability could be questioned given we're planning a 1:1 therapy, one hour a week for 7 weeks.<br>However, the intention is that this would reduce overall duration of time the patient is in the clinic. Targeted at current non-responders to the current treatment, so would likely go on to having complex health conditions/ NHS expense.<br><br>Using online platform (e.g. zoom) reduces cost. No clinic space required.<br>The feasibility trial is designed to determine if it is acceptable.
Restriction	No – this approach is too 'top-down' to be fitting with generating intrinsic motivation however the restriction of patients' exposure to interventions that engage extrinsic motivation would be beneficial.	
Environmental Restructuring	N/A	No
Modelling	Parents and therapists will be encouraged to model behaviours driven by intrinsic motivation, which will support the young person to learn vicariously.	Yes
Enablement	Using ACT to increase self-determination will hopefully help reduce barriers patients have so far found in their weight management.	Yes
	**Selected intervention functions:**<br><br>Modelling, enablement, training & education	

Step 6: Identify policy categories

Of the seven policy categories, as listed below in Table 2, use APEASE criteria to identify the categories best suited to deliver the identified intervention functions within the resource constraints of the work.

Table 3. Matrix of links between intervention functions and policy categories

Policy Categories	Intervention Functions								
	Education	Persuasion	Incentivisation	Coercion	Training	Restriction	Environmental restructuring	Modelling	Enablement
Communication/ Marketing									
Guidelines									
Fiscal									
Regulation									
Legislation									
Environmental/ social planning									
Service provision									

Table 4. examples of the policy categories

Policy category	Definition	Example
Communication/ marketing	Using print, electronic, telephonic or broadcast media	Conducting mass media campaigns
Guidelines	Creating documents that recommend or mandate practice. This includes all changes to service provision	Producing and disseminating treatment protocols
Fiscal measures	Using the tax system to reduce or increase the financial cost	Increasing duty or increasing anti-smuggling activities
Regulation	Establishing rules or principles of behaviour or practice	Establishing voluntary agreements on advertising
Legislation	Making or changing laws	Prohibiting sale or use
Environmental/social planning	Designing and/or controlling the physical or social environment	Using town planning
Service provision	Delivering a service	Establishing support services in workplaces, communities, etc.

Worksheet 6.

Intervention function	COM-B component	Potentially useful policy categories	Does the policy category meet the APEASE criteria in the context of improving paediatric patients' intrinsic motivation?
Education	Psychological Capability	Communication/marketing	No
		Guidelines	Yes
		Regulation	No
		Legislation	No
		Service provision	Yes
Training	Psychological Capability	Guidelines	Yes
		Fiscal measures	No
		Regulation	No
		Legislation	No
		Service provision	Yes
Modelling	Social Opportunity	Communication/marketing	No
		Service provision	Yes
Enablement	Reflective motivation	Guidelines	Yes
		Fiscal measures	No
		Regulation	No
		Legislation	No
		Environmental / Social planning	No
		Service provision	Yes
**Policy Category selected:** Guidelines & Service Provision			

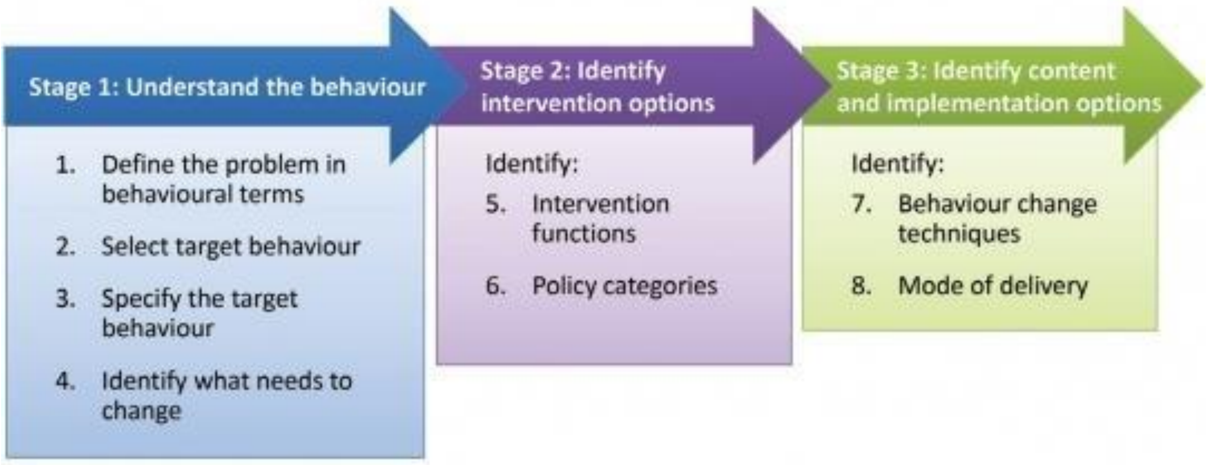

Stage 3: Identify content and implementation options.

Step 7: Identify behaviour change techniques.

Whilst the COM-B model traditionally leads us to utilise the behaviour change taxonomy of 93 individual behaviour change techniques (BCTs) covering all areas of COM-B (capability, opportunity and motivation), in this case the behaviour we are seeking to change is that of intrinsic motivation. For this, a specific set of 21 behaviour change techniques has been devised based on expert consensus (Teixera et al., 2020). As this tailored resource exists, we will continue the COM-B process but with reference to these behaviour change techniques instead, referred to as MBCTsBCTs (Motivational behaviour change techniques).

The suitability and potential efficacy of each identified BCT will be considered, guided by APEASE, to produce a final set of ~~BCTs~~BCTs for inclusion in the intervention.

We have also considered the BCTs needed to run the wider intervention, based on the area raised in the earlier stages of this behavioural analysis.

Worksheet 7a

**Intervention Function**	**COM-B Component**	**Most Recently used BCTs**	**Does the BCT meet the APEASE criteria (affordability, practicability, effectiveness/cost-effectiveness, acceptability, side-effects/safety, equity) in the context of raising patients' intrinsic motivation?**
**Education**	**Psychological capability Reflective motivation**	Information about social and environmental consequences	**Yes -** about the benefits of intrinsic motivation not weight loss
		Information about health consequences	**Yes -** about the benefits of intrinsic motivation not weight loss
		Feedback on behaviour	**Yes**
		Feedback on outcome of the behaviour	**Yes**
		Prompts/cues	**Yes**
		Self-monitoring of behaviour	**Yes**
		Biofeedback	Not practical to deliver

		Self-Monitoring of the outcomes of behaviour	Not relevant in this context
		Cue signally reward	Not relevant in this context
		Satiation	**Yes**
		Information about antecedents	**Yes**
		Reattribution	**Yes**
		Behavioural experiments	**Yes**
		Information about emotional consequences	**Yes -** about the benefits of intrinsic motivation not weight loss.
		Information about others' approval	**Unlikely to be effective in this context**
**Training**	~~**Physical capability**~~ **Psychological Capability Automatic motivation** ~~**Physical opportunity**~~	Demonstration of the behaviour	**Yes**
		Instruction on how to perform a behaviour	**Yes**
		Feedback on the behaviour	**Yes**
		Feedback on outcomes of behaviour	**Yes**
		Self-monitoring of behaviour	**Yes**
		Behavioural practice/rehearsal	**Yes**
		Biofeedback	Not practical to deliver.
		Self-monitoring of outcomes of behaviour	**Yes**
		Habit formation	**Yes**
		Habit reversal	**Yes**
		Graded tasks	**Yes if set collaboratively.**
		Behavioural experiments	Not relevant in this context,

		Mental rehearsals of successful performance	**Yes**
		Self-talk	**Yes**
		Self-reward	**Yes**
**Modelling**	**Automatic motivation**<br>**Social opportunity**	Demonstration of the behaviour	**Yes**
**Enablement**	**Psychological capability**<br>**Automatic motivation**<br>**Social opportunity**	Social support (unspecified)	**Yes – if in line with autonomy support**
		Social support (practical)	**Not likely to effective in this context**
		Goal setting (behaviour)	**Yes**
		Goal setting (outcome)	**Yes**
		Adding objects to the environment	**Not relevant**
		Problem solving	**Yes – promoting the person to problem solve, not problem solving for them**
		Action planning	**Yes**
		Self-monitoring of behaviour	**Yes**
		Restructuring the physical environment	**Not relevant**
		Review behavioural goals	**Yes**
		Review outcome goals	**Yes –**
		Social support (emotional)	**Yes – if in line with autonomy support**
		Reduce negative emotions	**Not likely to be effective**
		Conserve mental resources	**Not likely to be effective**
		Pharmacological support	**Not relevant**
		Self-monitoring of outcomes of behaviour	**Yes**
		Behavioural substitution	**Not likely to be effective**
		Overcorrection	**Not likely to be effective**
		Generalisation of a target behaviour	**Not likely to be effective**
		Graded tasks	**Yes – if self-set**

		Avoidance/reducing exposure to cues for the behaviour	**Not likely to be effective**
		Restructuring the environment	**Not relevant**
		Distraction	**Not likely to be effective**
		Body changes	**Not relevant**
		Behavioural experiments	**Yes**
		Mental rehearsal of the successful performance	**Yes**
		Focus on past success	**Yes**
		Self-talk	**Yes**
		Verbal persuasion about capability	**Not likely to be effective**
		Self-reward	**Yes**
		Behavioural contract	**Not likely to be effective**
		Commitments	**Not likely to be effective**
		Discrepancy between current behaviour and goal	**Collaboratively**
		Pros and cons	**Yes**
		Comparative imagining of future outcomes	**Yes**
		Valued self-identity	**Yes**
		Framing / reframing	**Yes - collaboratively**
		Incompatible beliefs	**Yes – but manner of delivery is important**
		Identity associated with changed behaviour	**Yes**
		Identification of self as a role model	**Yes**
		Salience of consequences	**Not likely to be effective**
		Monitoring of emotional consequences	**Yes**
		Anticipated regret	**Not likely to be effective**
		Imaginary punishment	**Not likely to be effective**
		Imaginary reward	**Not likely to be effective**
		Vicarious consequences	**Not likely to be effective.**

Worksheet 7b: Additional MBCTs applicable to this intervention

Label	Definition	Function	Description	Does the MBCT meet the APEASE criteria?
Autonomy-Support Techniques				Code:<br>Yes,<br>Not relevant in this context,<br>Unlikely to be effective in this context or<br>Not practical to deliver
MBCT1.	Elicit perspectives on condition or behaviour	Encourage exploration and sharing of perspectives on current behaviour (e.g. causes, perpetuating factors etc.).	Allows exploration of behaviour in more depth (self-knowledge), which can inform the programme and personal choices.	Yes
MBCT2.	Prompt identification of sources of pressure for behaviour change	Prompt identification of possible sources of external (or partially internalized) pressures and expectations and explore how they may relate to client's desired goals and outcomes.	Explores locus of causality and potential sources of external/introjected regulation and its consequences	Yes
MBCT 3.	Use noncontrolling, informational language	Use informational, non-judgmental language that conveys freedom of choice, collaboration, and possibility when communicating (avoiding constraining, pressuring, or guilt-inducing language). For example, use "might" or "could" instead of "should" and "must".	Avoids being a source of pressure or creating internal pressure, countering external locus of causality for actions.	Yes

MBCT 4.	Explore life aspirations and values	Prompt identification and listing of important life aspirations, values, and/or long-term interests and explore how changes in behaviour (or maintaining the status quo) could be linked to them.	Explores integrity and internal coherence between aspirations, values, and goals/behaviours, which can sustain autonomous regulation.	Yes
MBCT 5.	Provide a meaningful rationale	Prompt client to identify rationale for behaviour change and its maintenance that is tailored, explanatory, and personally meaningful or valuable.	Highlights and reinforces motives/reasons that could form the basis of autonomous motivation.	Yes
MBCT 6.	Provide choice	Provide opportunities to make choices from a collaboratively devised menu of behavioural options and autonomous goals. It includes the decision not to change, delay change, select focus/intensity of change, personally endorsed intrinsic goals and standards for success, including the timing or pace for certain outcomes.	Promotes personal input and ownership over behaviour change and responsibility through choice	Yes
MBCT 7.	Encourage the person to experiment and self-initiate the behaviour	Prompt the person to experiment and self-initiate (new) target behaviour that could be fun and enjoyable, is experienced as positive challenge, opportunity for learning or personal expression, and/or is associated with skill development, all of which provide experiential / immediate positive reinforcement".	Supports autonomous action via intrinsic motivation.	Yes
Relatedness-support techniques				

MBCT 8.	Acknowledge and respect perspectives and feelings	Provide statements of empathy and acknowledgment of the person's perspective, conflicts/ambivalence, distress, and negative affect (fear, confusion, etc.) and also expression of positive feelings when communicating with client (concerning the target behaviour, treatment, or other related matters).	Indicates attention and respect for the person's attitudes, thoughts, perceptions, and feelings, which creates an accepting and warm social environment.	Yes
MBCT 9.	Encourage asking of questions	Prompt the client to pose questions regarding their goals/behavioural progress.	Creates an open and collaborative relationship that promotes trust.	Yes
MBCT 10.	Show unconditional regard	Express positive support regardless of success or failure.	Demonstrates unconditional respect, care and support and promotes warm social environment.	Yes
MBCT 11.	Demonstrate/show interest in the person	Provide statements of interest and curiosity about the person's thoughts and perceptions, personal history and background, social context, life events, etc. when communicating.	Displays involvement, indicates to the person that their experiences and input are valued.	Yes
MBCT 12.	Use empathic listening	Demonstrate attentiveness to the client's responses (e.g. stay silent to allow the person to complete sentences), and provide reflective and summary statements when appropriate (directed at affect or content) when communicating. Prompt permission to	Creates open, collaborative relationship; promotes trust; Displays respect for the person.	Yes

		provide new information, guidance or advice		
MBCT 13.	Providing opportunities for ongoing support	Offer the person an appropriate venue and means to contact you in the event of difficulties or questions during the behaviour change process.	Shows care and personal involvement.	Not practical to deliver
MBCT 14.	Prompt identification and seek available social support	Prompt identification of sources of support for behaviour change (if relevant), acknowledge challenges in recruiting adequate support (autonomous vs controlled), and promote effective ways of seeking positive support.	Includes strategies that will help in feeling confident to overcome potential challenges and meet behavioural goal (e.g. information about available programmes, active involvement of others such as family members).	Yes
Competence-support techniques				
MBCT 15.	Address obstacles for change	Prompt identification of likely barriers to behaviour change, based on previous attempts, and explore how to overcome them (e.g. what may have worked in the past).	Increases confidence and reinforces existing skills.	Yes
MBCT 16.	Clarify expectations	Prompt statements of client's own expectations in terms of behaviour change (e.g. identify a clear goal or learning objective), both its experiential elements (process) as well as outcomes.	Provides structure and minimizes future failure (and perceived incompetence).	Yes

MBCT 17.	Assist in setting optimal challenge	Assist in identification of goals that are realistic, meaningful challenging, and achievable.	Provides structure and minimizes future failure (and perceived incompetence)	Yes
MBCT 18.	Offer constructive, clear, and relevant feedback	Provide relevant, tailored, non-evaluative feedback on goal/behavioural progress. This can include specific, process-focused feedback.	Provides encouragement and information to guide future behaviour.	Yes
MBCT 19.	Help develop a clear and concrete plan of action	Develop and provide summary of action plan to work toward a behavioural goal.	Provides structure, increases confidence, and minimizes future failure (and perceived incompetence).	Yes
MBCT 20.	Promote self-monitoring	Prompt monitoring of progress, skill level, or performance such as suggesting options for monitoring tools/means and metrics for success, including steps in the direction of behaviour change.	Provides structuring information that reinforces success and self-awareness.	Yes
MBCT 21.	Explore ways of dealing with pressure	Provide information to manage and limit effects of pressuring contingencies that would undermine competence such as extrinsic rewards, criticism, negative feedback.	Increase confidence to deal with sources of controlling pressure from others and themselves.	Yes

Step 7: Identify mode of delivery.

The final step involves considering the following in relation to intervention delivery: content, provider, recipients, intensity, duration and fidelity. The various potential modes of intervention delivery were considered using the APEASE criteria to assess the options that would be suitable within the constraints and resources of the feasibility trial.

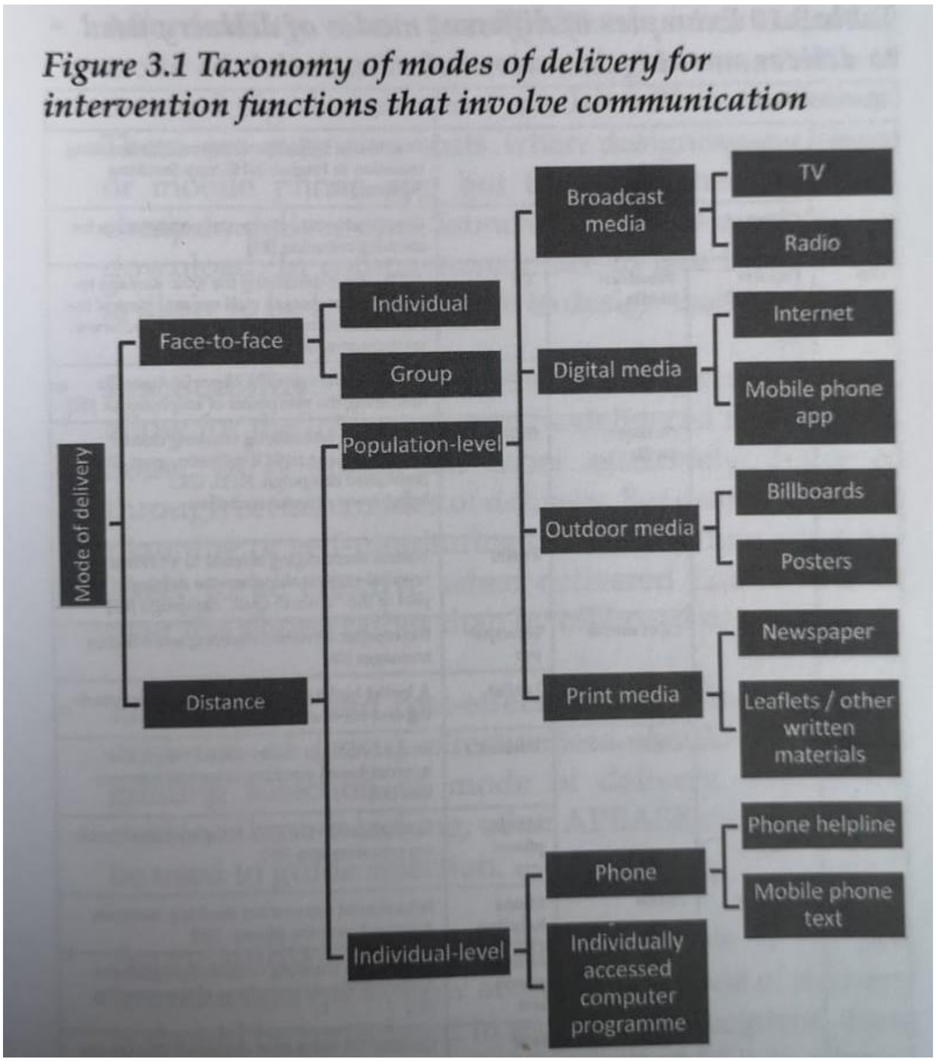

Figure 3.1 Taxonomy of modes of delivery for intervention functions that involve communication

Worksheet 8.

Mode of delivery				Does the mode of delivery meet the APEASE criteria in the context of increasing paediatric patients' intrinsic motivation?
Face-to-face	Individual			Yes – will need to consider the costing / cost-effectiveness as face-to-face is expensive
	Group			Yes
Distance	Population level	Broadcast media	TV	Not applicable
			Radio	Not applicable
		Outdoor media	Billboard	Not applicable
			Poster	Not applicable
		Print media	Newspaper	Not applicable
			Leaflet	Not applicable
		Digital media	Internet	Not applicable
			Mobile phone app	Not applicable
	Individual level	Phone	Phone helpline	Yes – partially if video calling. Calls could be used if preferred by patient, although much easier to share materials and build rapport using a platform such as zoom (or equivalent).
			Mobile phone text	The intervention is too complex to be delivered over text. Needs discussion between therapist and patient
		Individually accessed online programme		Yes – could be delivered as a remote programme, but patients would potentially lose out through not working with a clinician. Cost-benefit analysis required.

Table of changes

The table of changes is an iterative document that has been contributed to following PPI meetings and will be updated throughout the iterative development phase of the intervention.

Coding key:
IMP = Important behaviour change
EAU = Easy and uncontroversial
REP = Repeatedly
EXP = Experience (specify PPI, experts, literature)
NCON = Does not contradict experience or the guiding principles
NC = not changed (give a reason)
MoScoW = Must do, Should do, Could do, Would like to do

Aspect of the intervention	Negative Comments	Positive comments	Possible changes	Reason for change code	Agreed change	MoScoW (Must do, Should do, Could do, Would like to do)
Concept feedback						
Novel		The approach was novel and enabled them to think about their feelings in a way they had not before.				
Internal/self-motivation	PPI members explained repeated failed attempts at weight loss until they themselves felt they were ready. Our challenge is that the YP's have been referred, we need to tap into a way of getting them ready to start, from a place where they may not have chosen to be referred.	Thought to be v important for successful weight change. Not going to continue with an intervention that "makes them feel rubbish".<br><br>GB "Got to have that moment where you think I need to do this for me" and GT agreed need to "decide for myself" to	Consider methods to drive internal motivation including visualisation, clarification of values. Reduction of external pressures may give	IMP, EXP	Incorporate methods to drive internal motivation including visualisation, clarification of values.<br>Reduction of external pressures may give space for internal motivations.	Must do

		make the change and to stop thinking of healthy eating as a negative thing to be doing, instead framing it as a positive change.	space for internal motivations.			
Wholistic		This concept came up in both adult PPI sessions – the importance of seeing the whole person rather than purely focussing on what they eat. BC towards end felt the approach was "empowering, hopeful and for the whole person"<br><br>The lack of focus on specific food, and more about whole person and behaviour as a strong benefit.				
Flexible approach as opposed to strict rules.	For those with poor nutritional knowledge, more guidance may be required to establish a healthy diet.	PPI members reflected on their experience of wanting something more because it had been restricted- a flexible approach is preferred.		Exp. NCON	As the clinic already offers nutritional education we hope that patients have learnt during their initial 6-months at the COCO clinic. Dietary guidelines and calorie counting is also offered so this intervention seeks to take a different approach to support those for whom that approach has not worked.	
Individualised approach	The importance of making the intervention relevant to the individual's interests and motivations (gave example of his therapist using his interest in cars)		Develop individual rapport with patients enabling metaphors and examples to be tailored to their needs	EXP,	Therapists' confidence with the programme material will need to be considered when thinking about their ability to adapt interventions on the spot.<br>If possible, this would be the	Would like to do

					ideal, but feasibility will need to be reviewed.	
Logistical factors						
1-2-1 sessions		Felt that YP need to be a certain age before being able to share their feelings within a group				
Where to hold the sessions		As much choice given to the young people as possible.				
Parents joining in the sessions.	Young people may struggle to discuss how they feel in front of parents, especially before they have built rapport with the clinical team enough to feel safe to discuss their feelings.<br><br>Holding these discussions in front of family may be particularly sensitive if the young person feels their upbringing and/or home food environment has contributed to their weight problem.<br><br>Equally parents may have questions and experiences they want to share with the clincians but not in front of the young person.		Splitting sessions so part is done with the YP alone to develop trust and confidence to share with their PG<br>Or<br>Offering the young person the chance to invite their parent to join or not	REP, EXP, NCON	The young person will get the chance to invite their parent when they want to - session topics will be give a week ahead so young people can make a session by session choice. Currently, no session time has been allocated for parents-only, however this will be reviewed.	Should do
Default of inviting the parent to support	The parent may not be the most appropriate support, in some cases a grandparent, or other figure may be the child's key support.	As much choice given to the young person about the session is a good thing.	Young people being given the option to bring an alternative support person, not defaulting to always being the parents.	EXP, NCON	This option will be offered to patients, and continually reviewed.	Could do

Online video as the platform for therapy	But might create problems if nowhere to go at home without parents. Don't know who else is there to 'make sure they are saying the right things'.	Some cases zoom better, e.g. video off, may help to be relaxed in a comfortable place, more honest.				
Terminology						
Terminology – unhooking		The wording resonated – in particular 'unhooking' and 'hooked' was described to explain how they feel about being absorbed by darker thoughts, with strategies such as going for a walk being 'unhooking' they reflected.	offer several activities to try for each session as individual differences in how each activity will be viewed and received.	/		
The use of the term 'Choice'	Does choice have connotations of fault and blame? That 'choice' is a difficult word. As YP often told they have choices but often they are already made (by adults, socioeconomic position, genetics - CG). HH "YP may not yet understand that they are in control of their behaviour." That they might not realise they are making choices. JW – remembered feeling that she didn't understand her emotions "never mind that I had a choice". Linked to DF (Thurs11th) who felt when muddled, she didn't feel she had a		think about how to use the word choice so it avoids negative feelings surrounding not having made good ones previously. Raising awareness of the choices we make all the time – some YP may not be aware or even able to make 'good' choices when feeling emotional and pressured.		Work to be done on explaining the concepts, before then introducing choice as something we would work on giving the YP. Rather than inferring that they have always had choice and have been taking the wrong choice up until this point.	

	choice in the way she behaved around food.					
Using the term 'mindfulness'	The term 'mindfulness' is overused especially during school and when stress is discussed. It makes people stop listening as it feels like more of the same.	Some adolescent PPI members had positive experiences and a regular mindfulness practice, therefore connecting that the activities were mindfulness allowed them to build on their skills<br><br>Important to not underestimate young people's interest and engagement:	Using the term 'awareness' i.e. bringing your awareness to certain experiences, rather than mindfulness may help tap into the beneficial effects of mindfulness without the cliché preconceptions.	EAU, REP, EXP NCON	Both terms will be used and the preferred language will be discussed with each young person. This is an area for continued monitoring and can be changed if there is a clear pattern.	Could do
Specific activities						
Three mountain metaphor	The metaphor simplifies the challenges of the journey and the external pressures including peer pressure and technology that impact the journey<br><br>one person felt it could be viewed competitively, so suggested making sure it wasn't viewed as a race or competition	The collaborative aspects of the therapy relationship ae clear, the approach of tackling these problems together, with lots of emphasis on how the young person is involved in shaping this process.<br><br>it was felt this approach felt "inviting". "Invitation to come on a joint journey"<br><br>"makes them know it's what they want rather than being told to do something."	Ensure that the diagram of the mountain includes notable up's and down's and discuss them in the explanation of the metaphor	EAU, EXP, NCON	Yes	Should do
Lottery	YP's weren't as keen on the lottery example as made them feel selfish.					

	Found it hard to decide how to see yourself.					
Inflatable ball activity		The activity facilitated conversation; it was easier to discuss having done the activity together.<br><br>People then built on the metaphor, continuing to use it in new ways to explain how they felt.<br><br>"Feel like you described my teenage years" "a new way to consider this".<br><br>Strength in that the conversation evolved, with people still using the metaphor to explain how they felt in other situations				
The choice point video	The video simplifies the decision to make the life-enhancing decision, rather than the habitual non-healthful behaviour.<br><br>Video features a very slim woman with protruding collarbones with PPI members found drew their attention.<br><br>Video only features a woman.	The video captured attention and offered a concise snapshot of what was perceived as an interesting and novel approach. Left people wanting to know more about the approach.<br><br>The video offered hope that there was a solution not just to their weight but to how they were feeling more broadly. Gave them a sense that they didn't need to feel stuck and left them feeling more positive.	Caveat the video with the understanding that this is a simplified version of what is actually going on.<br><br>There are two versions of the video, so ensuring the version with the slim woman is not used.	Exp. NCON		Should do

	Some of the language/issues were not necessarily the right focus for YP (e.g. financial worries).	Discussions were held about how the video was targeted at adults, but that this wasn't necessarily a negative thing as for many YP's it would be empowering to know that they were being treated as adults.<br><br>"Got the point across without being super cringy".				
Visualising your mind as a character	Not all people enjoyed trying to draw and visualise their minds. One person who was less keen on the activity felt their mind often encouraged them to engage in the health beneficial behaviours.	Some people found the idea that our mind tells us the same repeated stories useful and enjoyed reflecting on this.<br><br>Members explained battling with the two sides of their mind	Assessing where the YP is at individually may help to understand whether this is a useful activity for them	EXP,	developing the baseline that these are all things to experiment with/try. Some will feel helpful, others may not, and that is okay – we can just keep working with/building on the things that feel they work for that individual. Not everything will work for everyone.	Could do
